# Social Involvement Modulates the Response to Novel and Adverse Life Events in Mice

**DOI:** 10.1371/journal.pone.0163077

**Published:** 2016-09-15

**Authors:** Luca Colnaghi, Kelly Clemenza, Sarah E. Groleau, Shira Weiss, Anna M. Snyder, Mariana Lopez-Rosas, Amir A. Levine

**Affiliations:** 1 Department of Psychiatry, College of Physicians and Surgeons, Columbia University, New York, New York, United States of America; 2 Department of Neuroscience, Centre for Addiction and Mental Health, Toronto, Ontario, Canada; 3 Department of Neuroscience, McMaster University, Toronto, Ontario, Canada; 4 Division of Child and Adolescent Psychiatry, New York State Psychiatric Institute, New York, New York, United States of America; Klinikum der Johann Wolfgang Goethe-Universitat Frankfurt, GERMANY

## Abstract

Epidemiological findings suggest that social involvement plays a major role in establishing resilience to adversity, however, the neurobiology by which social involvement confers protection is not well understood. Hypothesizing that social involvement confers resilience by changing the way adverse life events are encoded, we designed a series of behavioral tests in mice that utilize the presence or absence of conspecific cage mates in measuring response to novel and adverse events. We found that the presence of cage mates increased movement after exposure to a novel environment, increased time spent in the open arms of the elevated plus maze, and decreased freezing time after a foot shock as well as expedited fear extinction, therefore significantly changing the response to adversity. This is a first description of a mouse model for the effects of social involvement on adverse life events. Understanding how social involvement provides resilience to adversity may contribute to the future treatment and prevention of mental and physical illness.

## Introduction

In life, we tend to experience situations differently in the presence or absence of social involvement. This is especially important in relation to how we perceive adverse life events, since negative experiences play a determining role in the pathophysiology and time of onset of mental illness [1, 2],. However, not all people who experience adversity go on to suffer negative physical and emotional consequences. Among the various genetics and environmental factors that could mitigate the potential negative outcomes of exposure to adverse events is social involvement. For example, Stockdale et al.[3] found that living conditions with greater social involvement, such as neighborhoods with a higher average household occupancy and churches per capita, were associated with a lower incidence of mental disorders. Moak and Agrawal [4] found that increased perceived interpersonal social support ameliorated the pathogenic influence of exposure to traumatic life events on psychopathology, enhancing individuals' general mental and physical well-being, both in daily life and upon exposure to negative life events. Finally, Brewin et al. [5] and Brackbill et al. [6] found that low social support is one of the most common post-event risk factors for developing post-traumatic stress disorder. Together, these findings support the idea that the response to negative experiences may be affected by social involvement. Despite the important implications of these epidemiological findings, how social involvement confers protection against negative experiences remains poorly understood, and the neuronal mechanisms underlying this effect remain to be elucidated. In this work we set out to examine, in a mouse model, whether social involvement modulates the response to an adverse environment or event, and whether it changes the way these events are encoded in the brain to affect behavior. We therefore examined whether mice respond to adverse experiences differently in the presence or absence of cage mates in several behavioral tests: (a) exposure to a novel well-lit new environment; (b) an elevated plus maze (EPM) test that followed a habituation trial to the open field in the presence or absence of cage mates; (c) exposure to a strong foot-shock during contextual fear conditioning. We found that social involvement is associated with increased exploration as measured in increased movement when mice are exposed to a new environment, increased time spent in the open arms of the EPM after an obligatory exposure to the EPM open arm, and decreased fear memory as measured in a reduction in freezing time in response to a foot shock as well as faster fear extinction. This mouse model demonstrates that social involvement plays a role in mitigating adverse life events.

## Material and Methods

### Animals

Male C57BL6 mice between 10 and 14 weeks old (The Jackson Laboratory, Bar Harbor, ME) were used for all experiments. Mice were kept in clear plastic cages with ad libitum food and water. All animal procedures described were executed in accordance with National Institute of Health regulations. The protocol was approved by the Institutional Animal Care and Use Committees of Columbia University and the New York State Psychiatric Institute. Except for mice who were used in the Phenotyper experiment, all mice were housed in groups of 5 throughout the experiments.

### Behavioral Testing

#### PhenoTyper Experiment

Behavioral tracking was carried out in an automated home-cage environment, the PhenoTyper (45 cm x 45 cm x 55 cm; Noldus Technology; Leesburg, Virginia). PhenoTyper cages consist of transparent walls, a sipper water bottle, a feeder, and a stationary shelter. Mice were split into two cohorts and placed in the Phenotyper arena alone (“Singles” Cohort) or in pairs (“Pairs” Cohort). Mice were placed in the phenotyper during the light phase, under bright lighting conditions, and tracked using a digital video camera and infrared lighting. Distance travelled (measured in cm) was acquired using EthoVision software (Noldus Technology; Leesburg, Virginia) over a 5-day period.

#### Elevated Plus Maze Experiment

The classical elevated plus maze was modified to test the role of social interactions in modulating anxiety response. On day one, access to the closed arms was blocked by a barrier. Mice were split into two cohorts and placed on either open arm of the elevated plus maze apparatus, placed either alone (“Singles” Cohort) or in groups of three cage mates (“Groups” Cohort). Mice were left on the open arms for 5 minutes and then returned to their home cage. The procedure was repeated on Day 2. On Day 3, mice were subjected to the classical elevated plus maze experiment [7]. All mice were scored in the absence of conspecifics. Time spent in the open and closed arms was timed, and entries into open and closed arms were counted.

#### Contextual Fear Conditioning Experiment

The classical contextual fear experiment was modified to examine differences in fear conditioning in the presence or absence of conspecific cage mates. The conditioning chamber (Med Associates) was fitted with a box constructed with clear plastic walls and an open top. This box was designed to house two additional mice such that they are visible to the mouse undergoing foot shock, but would be physically isolated from the electric grid. We refer to these mice as “spectators”.

On Day 1, mice were split into two cohorts and placed in the chamber either alone (“Singles” Cohort) or in the presence of two spectator mice from the same home cage (“Groups” Cohort). 2 minutes after placement mice were exposed to the unconditioned stimulus, 1.5 mA of continuous foot shock for 2 seconds. After an additional 30 seconds in the chamber, mice were returned to their home cage. Conditioning was assessed 24 hours later on Day 2. Conditioning was assessed by scoring freezing behavior, which was defined as the complete lack of movement, in intervals of 0.5 seconds. Contextual fear conditioning was assessed for 2 consecutive minutes in the chamber in which the mice were trained. All mice were scored in the absence of spectators.

#### Fear Extinction Paradigm Experiment

On the training day, mice were placed alone in the conditioning chamber (Med Associates) for 2 minutes before the onset of the conditioned stimulus (CS), a tone, which lasted for 30 seconds at 80 dB. For the experimental group, the last 2 seconds of the CS was paired with the unconditioned stimulus (US), 1.5 mA of continuous foot shock. After an additional 30 seconds in the chamber, the mouse was returned to its home cage. Cued and Contextual Fear Conditioning was assessed for 2 minutes in the chamber in which each mouse was trained. 30 seconds after the mouse placed in the chamber, the mouse was presented with the conditioning tone for 60 seconds. After an additional 30 seconds in the chamber, the mouse was returned to its home cage. 24 hours after the measure of fear conditioning, the mice underwent a fear extinction trial. Mice were either placed in the chamber alone (“Singles Conditioning/Singles Extinction” Cohort) or in groups consisting of five or three cage mates (“Singles Conditioning/Group Extinction” Cohort). 30 seconds after the single mouse or grouped mice were placed in the chamber, they were presented with the conditioning tone for 30 seconds. This cycle was repeated 4 times. 24 hours after fear extinction trial, Cued and Contextual Fear conditioning was assessed by placing the mice from both cohorts alone in the chamber in which the mice had undergone the group or individual extinction procedure and measuring their freezing time. For the control group, mice followed the same sequence of events, with the exception that there was no shock administered at the last 2 seconds of the CS on Day 1.

### Statistical Analysis

For all parameters (distance travelled centimeters (cm), time spent moving in seconds (s), velocity (cm/s), number of shelter exits, and freezing; paired and single mice were compared using a two-tailed t-test. Significant differences were defined by P-values (p≤0.05).

## Results

### Social involvement increases the time spent moving in a novel environment

**Fig 1:**
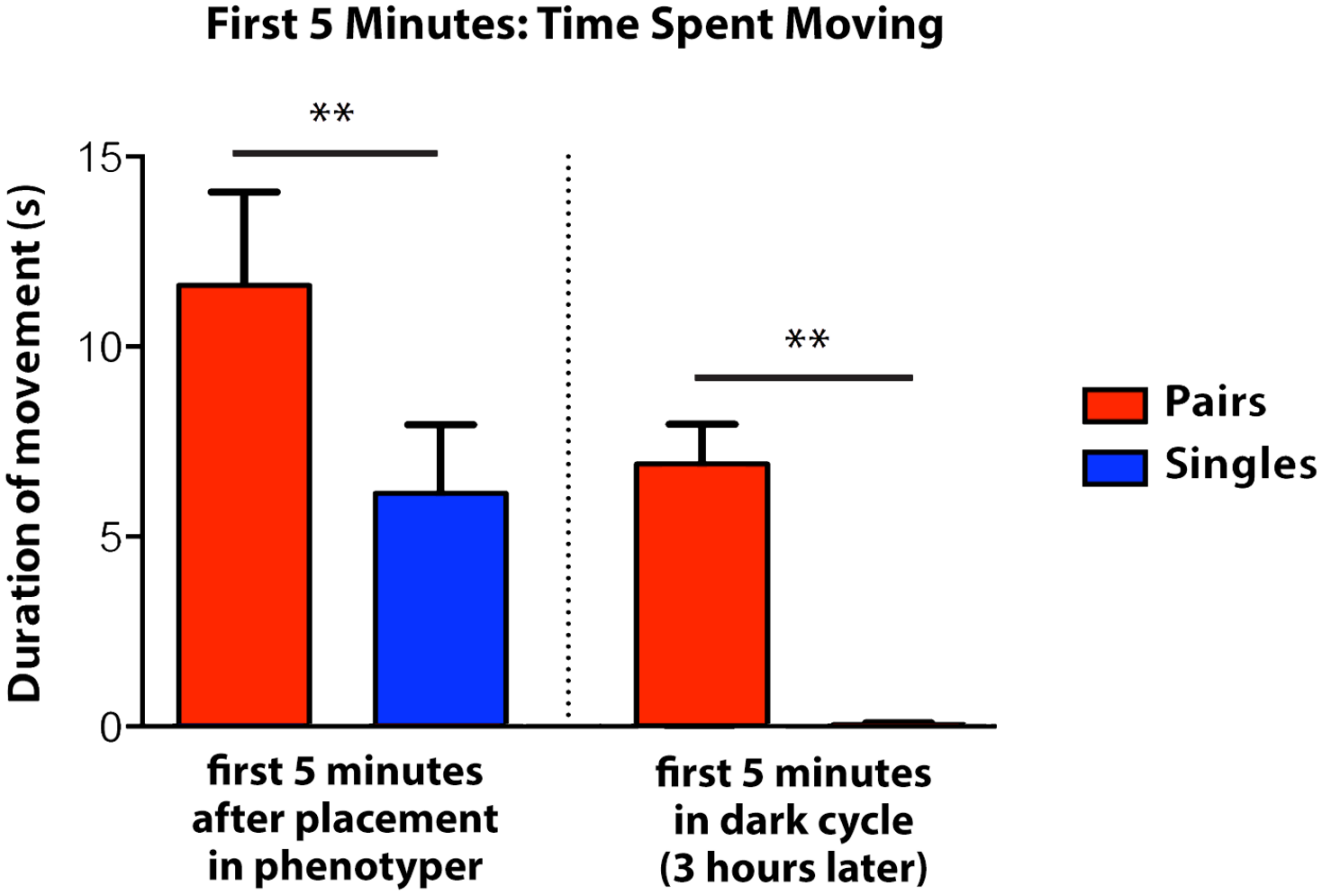
Time spent moving during first 5 minutes of light and dark cycles. Values represent time spent moving in the Noldus Phenotyper for the first five minutes after placement in the Phenotyper and the first five minutes of the dark cycle in groups of two (Pairs, red column) or alone (Singles, blue column). All values represent mean ± s.e.m. **P < 0.001, two-tailed *t* test (N = 12).

As a first step in exploring a possible role of social environment in modulating the response to adverse events, we began by asking, how does the presence of conspecific cage mates affect the response to a novel, well-lit (therefore aversive) environment? To address this, we took advantage of the Noldus Phenotyper system that allows us to track pairs of mice in a common arena. Mice were split into two cohorts and placed during the light cycle in a new, well-lit environment either individually (“Singles” Cohort) or in pairs (“Pairs” Cohort), and their behavioral response was recorded in the first five minutes after placement in the new arena. Measures of movement from the first five minutes after placement reflect the very immediate response to a somewhat adverse new setting. Paired mice demonstrated an increase in the distance traveled compared with single mice (Fig 1), suggesting that the presence of a cage mate is linked to increased exploration, potentially by reducing the anxiety associated with a novel surrounding. Next, we decided to test the effect of social interactions during the dark cycle, when mice are usually more active. To this end, following the recording during the light cycle, we left the mice in the arena until the beginning of the dark cycle, three hours later, and measured again the first 5 minutes of the new cycle. We found that in the dark cycle, several hours after placed in the arena, mice displayed increased activity when placed in pairs compared to single mice.

### Social involvement increases time spent in the open arms of the elevated plus maze (EPM)

**Fig 2:**
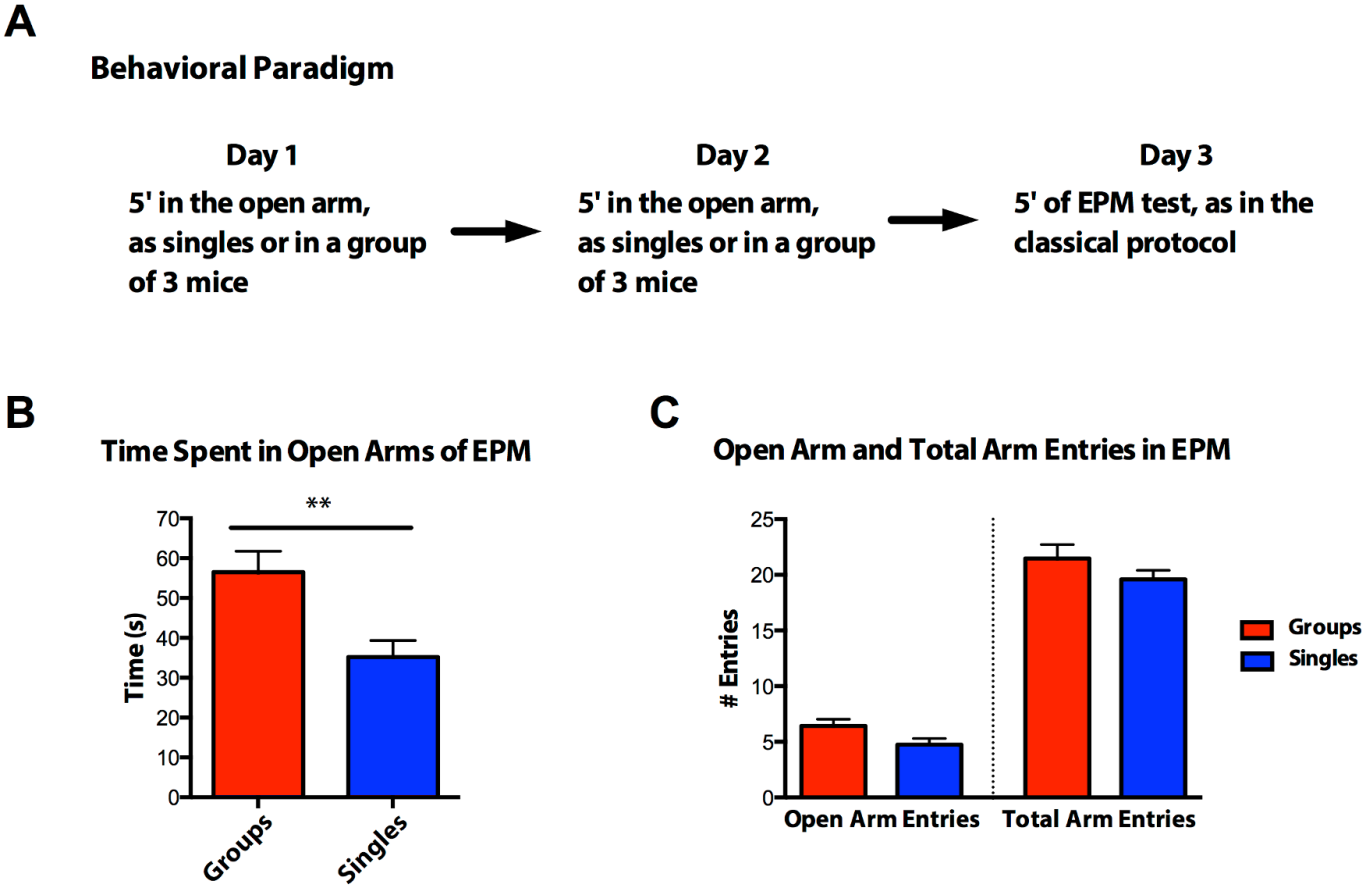
Time line of modified EPM test. (A) Diagram of behavioral paradigm. (B) Values represent time spent on the open arms during the test session in groups of three (Groups, red column) or alone (Singles, blue column). All values represent mean ± s.e.m. *P* = 0.0037, two-tailed *t* test (N = 15). (C) Values represent number of entries into the open arms, *P >* 0.05; and total entries (open+closed arms), *P >* 0.05; during test session either in groups of three (Groups, red column) or alone (Singles, blue column). All values represent mean ± s.e.m., two-tailed *t* test (N = 15).

To study further the link between the presence of conspecific cage mates and aversive environments, we modified the EPM test, a commonly used paradigm to test anxiety in mice, to assess longer–term effects of an exposure to anxiety provoking environment. For this purpose we performed a 2-tiered experiment, an exposure phase followed by a recording phase. For the exposure stage the arms of the EPM were closed off and the mice were placed in one of modified the open arms: this serves as an anxiety-provoking environment as mice are aversive to elevated and open spaces. Mice were placed in the modified open arms of the EPM apparatus either alone (“Singles” Cohort) or in groups of three (“Groups” Cohort), for two sessions (one per day) lasting 5 minutes each. 24 hours after the second session, mice performed the classical EPM test alone (Fig 2A). We found that mice that were placed in the open arms during the habituation sessions as a group spent more time in the open arms compared to mice that were placed in the obligatory open arms of the EPM alone (Fig 2B, without differing in the number of open or total arm entries (Fig 2C)). This finding suggests that being in the presence of cage mates during exposure to an anxiogenic environment decreases anxiety behavior without affecting overall number of arm entries when later placed alone in the EPM maze.

Together, the results reported in Figs 1–2 provide evidence that social involvement changes both the immediate as well as future reactions to an anxiety-provoking environment.

### Social interactions and highly aversive experiences

**Fig 3:**
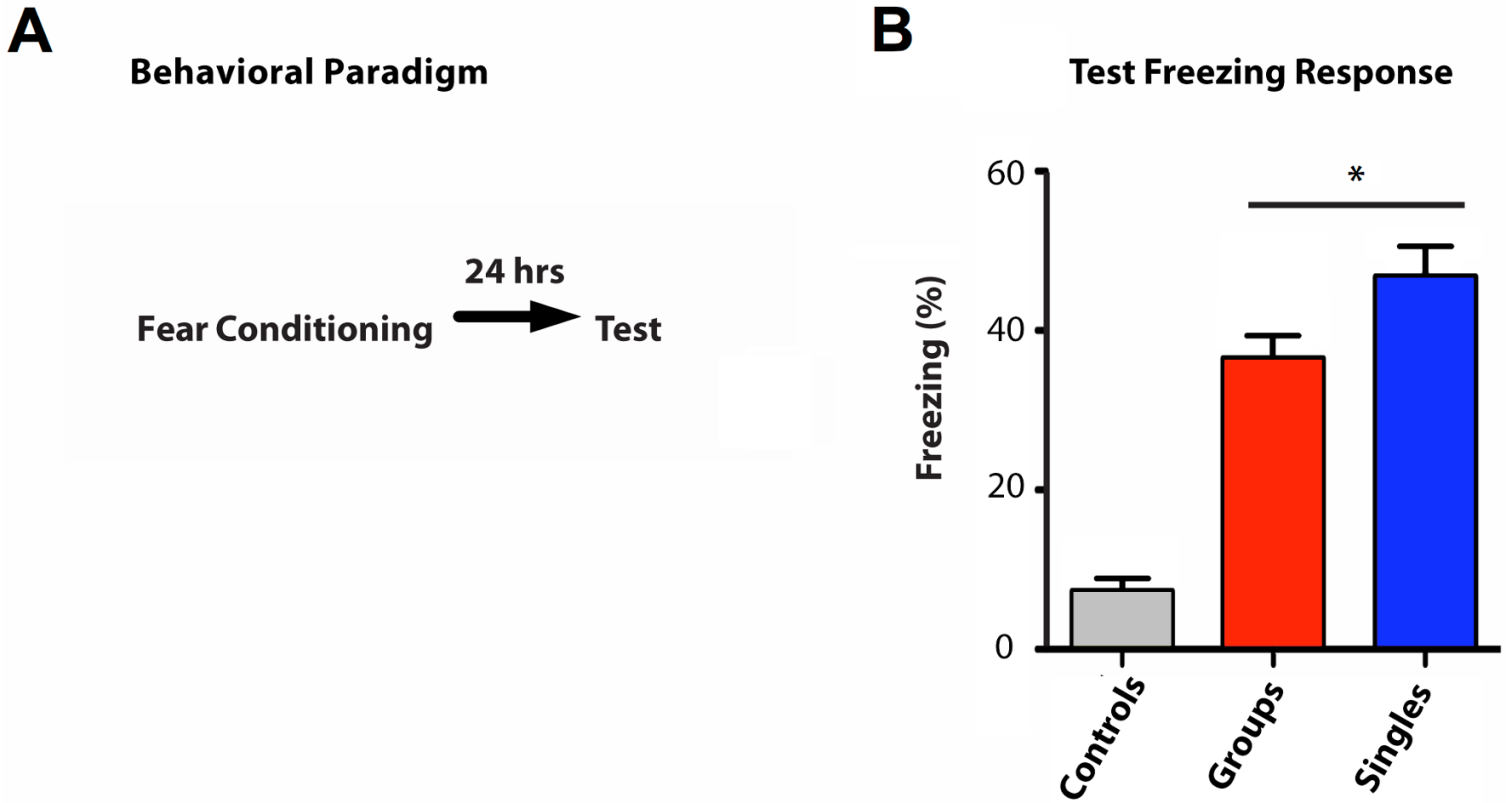
Time line of contextual fear conditioning. (A) Diagram of behavioral paradigm. (B) Values represent percentage of freezing for mice that experienced the shock in the presence of two spectators (Groups, red column) or alone (Singles, blue column) when re-exposed to the conditioning chamber, compared to control mice (Controls, grey column). All values represent mean ± s.e.m. *P = 0.027, two-tailed *t* test (N = 20).

Next, we asked whether social involvement modulates the effect on highly aversive experiences. To answer this question we tested whether mice encode fear memory of a strong foot shock differently in the presence or absence of their conspecific cage mates. Mice were placed in the conditioning chamber either alone (“Singles” Cohort), or in the presence of two spectators (“Groups” Cohort) (Fig 3). Spectators were housed in a clear plastic box inside the conditioning chamber so that they were visible to the mouse undergoing foot shock but were did not experience the shock themselves. We found that 24 hours after the foot shock was administered, when re-exposed to the same environment, the mice shocked in the presence of the spectators displayed less freezing compared to the mice shocked alone (Fig 3).

**Fig 4:**
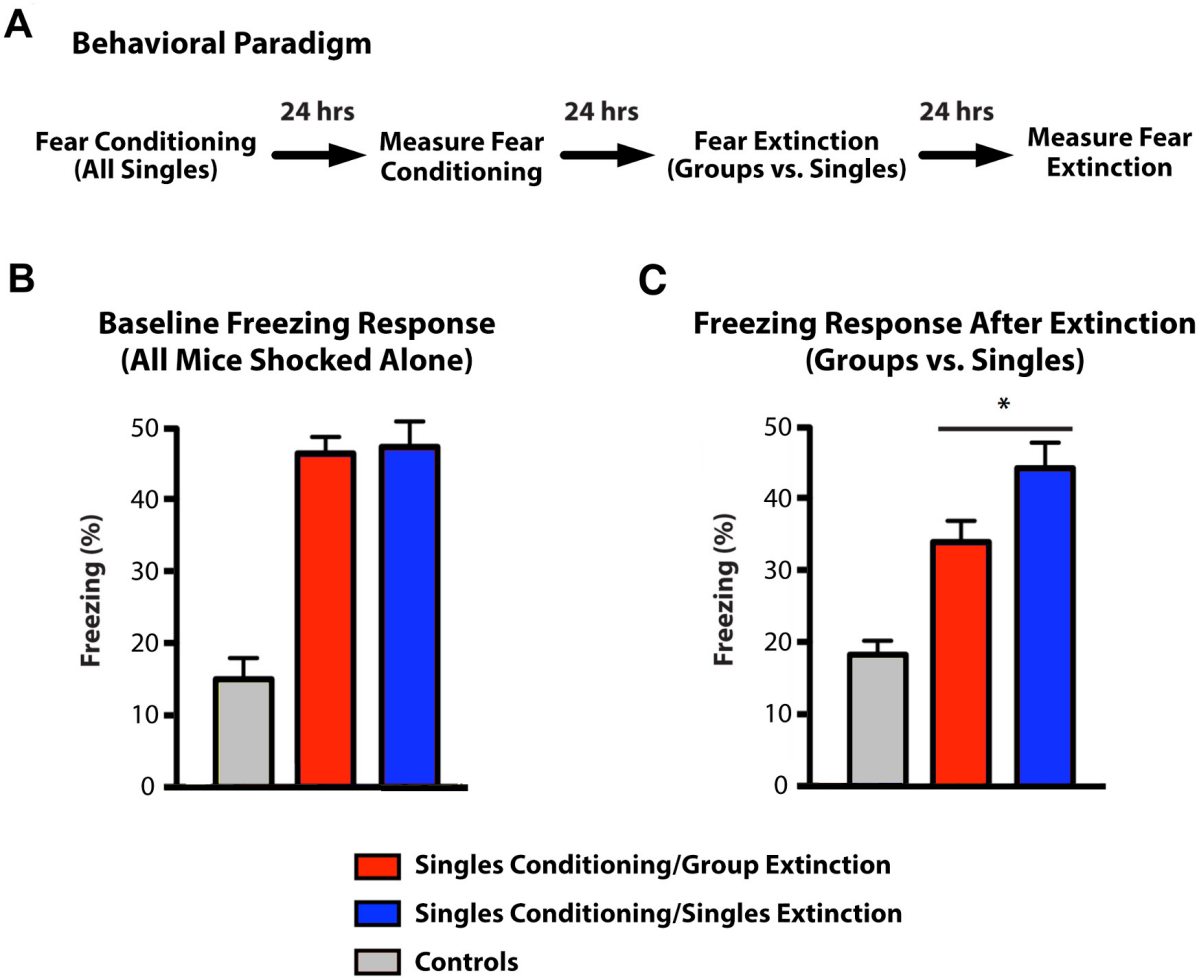
Fear extinction in groups of five versus alone. (A) Diagram of behavioral paradigm. (B) After fear conditioning, values represent percentage of freezing time for mice from both cohorts first exposed as singles, compared to non-foot shocked controls. (C) After fear extinction, values represent percentage of freezing time for mice when re-exposed to the conditioning environment as a group (Singles Conditioning/Singles Extinction, red column) or alone (Singles Conditioning/Group Extinction, blue column), compared to non-foot shocked controls. All values represent mean ± s.e.m. * P = 0.014, two-tailed *t* test (N = 15).

Next, we asked whether social interactions might also affect fear extinction in the aftermath of an exposure to a strong foot-shock. First, a foot shock was administered to all mice as singletons. 24 hours after contextual fear conditioning, the mice were separated into two cohorts and individually re-exposed to the conditioning chamber. The time spent freezing was assessed and as expected, the two cohorts did not show any significant differences in freezing (Fig 4B). 24 hours later, we performed an extinction trial. We re-exposed the mice to the conditioning chamber in groups of five cage mates (“Singles Conditioning/Group Extinction” Cohort) or alone (“Singles Conditioning/Singles Extinction” Cohort). 24 hours after the extinction trial the mice were individually re-exposed to the conditioning environment and freezing time was measured (Fig 4C). We found that when the mice were shocked alone, but then re-exposed to the chamber with their cage mates, they displayed increased extinction and exhibited less freezing than mice that underwent extinction trial alone (Fig 4). When three cage mates were present for the extinction as opposed to five, there was no difference between grouped and single mice, suggesting that the effect of social proximity on extinction is facilitated by a larger minimum number of conspecifics than the effect of cage mage conspecifics on the formation of fear memory (see S1 Fig).

Together, these results suggest that social involvement plays a role in decreasing fear memory in the aftermath of a highly adverse event, and moreover, if the highly adverse event has already occurred, exposure to the adverse environment in a group hastens extinction, albeit the extinction effect is facilitated in by the presence of additional cage mates.

## Discussion

Many organisms possess neuronal systems that ascribe a feeling of safety to social involvement. These systems have most likely evolved in the context of a survival advantage of being in the proximity of others who can inform and protect the individual against adverse events [8]. Thus a stimulus can be coded differently when one is alone or when one is in the company of other individuals. This means that social involvement could serve to mitigate the perception and processing of adverse events. Up until now, however, most of the work in this field using rodents has concentrated on the effect of social involvement on the spectators who witness conspecifics in distress. For example, studies have described how rats express behavioral and physiological responses to conspecific anguish such as cage fighting [9] or electric shock [10–14]. While these experiments suggest that empathy occurs in the social lives of rodents, little is known regarding how social interactions affect the processing of adverse events. Here, we describe that social involvement during a negative event plays a mitigating role during the actual event as well as in its aftermath. We found this to be the case in exposure to a novel or mildly adverse stimulus, where paired mice showed increased movement in a novel environment as well as increased time spent in the open arm following an exposure to the open arms of the EPM in a group. There is also an effect of cage mates on the encoding of fear memory as well: mice showed a decreased freezing time and a faster extinction rate when in a group vs. alone. Our findings are consistent with a study showing that following foot shock, rats showed attenuated stress-induced hypothermia and general behavioral response when together with a conspecific [15]. Moreover, we found that in mice social involvement increases physical activity and exploration (see companion paper Groleau et al 2016), further suggesting a close link between social interaction and anxiety. Together, our studies and the work of others [15] implicate that social involvement in rodents may regulate the response to adversity.

### Molecular mechanisms that may link social involvement to modulation of aversive events

Throughout the animal kingdom behaviors are modulated by social interaction. For example, in bacteria, in response to fluctuations in cell population density, a communication mechanism called quorum sensing causes changes in gene expression at the population level, to upregulate coordinated physiological activities such as motility and reproduction, that are specifically adapted to the social context. This social behavior is responsible for bacterial phenomenon such as biofilm formation, antibiotic production, and certain kinds of toxigenicity [16].

Another example of the relationship between social context, behavior and genes is provided by the songbird zebra finch (*Taeniopygia guttata*). Songbirds communicate between individuals through songs, which not only trigger several behavioral responses but also induce expression of genes in the brain. For instance, the immediate early gene *egr-1* is induced by the singing of another bird in a specific auditory region of the brain; however, the level of its induction depends on the presence of other conspecifics and can be correlated with the differential behavioral response to the same acoustic stimulus as a function of social context [17]. This suggests that the behavioral response to a stimulus depends on whether the subject experienced it alone or with others and that it is modulated by gene expression.

Immediate early genes such as egr-1, fos and jun are also induced by aversive experience and there is already evidence that, in rodents, at least one on them, fos, can be modulated by social context. Kikyokawa et al. found that in fear-conditioned rats, Fos expression in response to the fearful context was modulated by the presence of conspecifics [15]. It would therefore be of interest for future work to analyze whether activation of egr-1 and other immediate early genes, beside Fos, at the time of the aversive event is modulated by the presence of other mice. This class of genes regulates the transcription of hundreds of other genes and their effects on the physiology of cells are profound. Therefore understanding the modulation of immediate early genes by social involvement could be a crucial step towards our understanding of the molecular processes behind the effect of social involvement on aversive experiences, and it could help advance therapeutic interventions that will mitigate the affect of adversity on health.

## Conclusions

Epidemiological studies clearly describe a role for social involvement in promoting health and longevity, and mitigating the effect of adversity, however, the molecular bases underlying this effect is not known. Our results emphasize that social context modulate fear learning and processing in mice, providing a mouse behavioral model in which to study the neuronal circuits and molecular processes that are regulated by the presence of others and which play a role in the encoding and processing adverse stimuli as well as fear memory. Understanding these circuits better may lead to advancements and new therapeutics to target disorders such as PTSD and anxiety.

## Supporting Information

S1 Fig Fear extinction in groups of three versus alone. (A) After fear conditioning, values represent percentage of freezing time for mice from both cohorts first exposed as singles, compared to non-foot shocked controls. (B) After fear extinction, values represent percentage of freezing time for mice when re-exposed to the conditioning environment as a group (Singles Conditioning/Singles Extinction, red column) or alone (Singles Conditioning/Group Extinction, blue column), compared to non-foot shocked controls. All values represent mean ± s.e.m. * P = 0.811, two-tailed *t* test (N = 12).
(PDF)
