# Supplementary material for: Social Involvement Modulates the Response to Novel and Adverse Life Events in Mice
Source: PLoS One. 2016 Sep 15;11(9):e0163077. doi: 10.1371/journal.pone.0163077 (PMC5025180; doi:10.1371/journal.pone.0163077)

Cued and Contextual Fear Conditioning: Extinction as Singles vs. Groups of 3

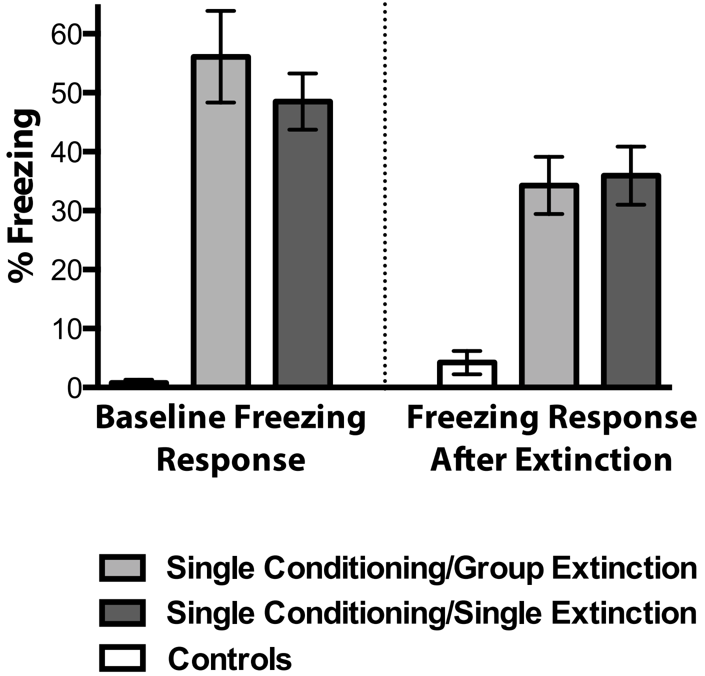

Supplement: S1 Fig — (A) After fear conditioning, values represent percentage of freezing time for mice from both cohorts first exposed as singles, compared to non-foot shocked controls. (B) After fear extinction, values represent percentage of freezing time for mice when re-exposed to the conditioning environment as a group (Singles Conditioning/Singles Extinction, red column) or alone (Singles Conditioning/Group Extinction, blue column), compared to non-foot shocked controls. All values represent mean ± s.e.m. * P = 0.811, two-tailed t test (N = 12). (PDF) [file pone.0163077.s001.pdf]
